# Identification of the *Mycobacterium ulcerans* Protein MUL_3720 as a Promising Target for the Development of a Diagnostic Test for Buruli Ulcer

**DOI:** 10.1371/journal.pntd.0003477

**Published:** 2015-02-10

**Authors:** Anita Dreyer, Katharina Röltgen, Jean Pierre Dangy, Marie Thérèse Ruf, Nicole Scherr, Miriam Bolz, Nicholas Jay Tobias, Charles Moes, Andrea Vettiger, Timothy Paul Stinear, Gerd Pluschke

**Affiliations:** 1 Swiss Tropical and Public Health Institute, Molecular Immunology, Basel, Switzerland; 2 University of Basel, Basel, Switzerland; 3 Department of Microbiology and Immunology, University of Melbourne, Parkville, Australia; University of Tennessee, UNITED STATES

## Abstract

Buruli ulcer (BU) caused by *Mycobacterium ulcerans* is a devastating skin disease, occurring mainly in remote West African communities with poor access to health care. Early case detection and subsequent antibiotic treatment are essential to counteract the progression of the characteristic chronic ulcerative lesions. Since the accuracy of clinical BU diagnosis is limited, laboratory reconfirmation is crucial. However, currently available diagnostic techniques with sufficient sensitivity and specificity require infrastructure and resources only accessible at a few reference centres in the African endemic countries. Hence, the development of a simple, rapid, sensitive and specific point-of-care diagnostic tool is one of the major research priorities for BU. In this study, we have identified a previously unknown *M. ulcerans* protein, MUL_3720, as a promising target for antigen capture-based detection assays. We show that MUL_3720 is highly expressed by *M. ulcerans* and has no orthologs in other prevalent pathogenic mycobacteria. We generated a panel of anti-MUL_3720 antibodies and used them to confirm a cell wall location for MUL_3720. These antibodies could also specifically detect *M. ulcerans* in infected human tissue samples as well as in lysates of infected mouse footpads. A bacterial 2-hybrid screen suggested a potential role for MUL_3720 in cell wall biosynthesis pathways. Finally, we demonstrate that a combination of MUL_3720 specific antibody reagents in a sandwich-ELISA format has sufficient sensitivity to make them suitable for the development of antigen capture-based diagnostic tests for BU.

## Author Summary

According to the recommendations of the World Health Organization, the clinical diagnosis of BU should be reconfirmed by at least two laboratory techniques. However, out of the four currently available tests, three (PCR, histopathology and cultivation of *M. ulcerans*) can only be performed at centralized reference laboratories; the fourth (microscopic detection of acid fast bacilli) lacks the required sensitivity and specificity. Therefore, a simple tool for early diagnosis of the disease, which can be implemented in rural health care facilities of the endemic countries, is of urgent need. In this study we aimed at the identification of *M. ulcerans* proteins as potential targets for the development of a simple and rapid diagnostic antigen detection assay. Among 36 proteins, MUL_3720 best met the predefined criteria of being highly expressed by *M. ulcerans* and not having orthologs in other pathogenic mycobacterial species prevalent in the endemic regions. Here we generated monoclonal and polyclonal antibodies against this protein and carried out pilot studies for the development of an antigen capture-based diagnostic test.

## Introduction

Buruli ulcer (BU) is a neglected mycobacterial skin disease, reported from tropical and subtropical countries world-wide with highest incidence rates in Western Africa [1]. Populations in rural areas with limited access to health facilities are most affected and often seek medical advice at late disease stages [2]. Advances in the clinical management of BU have shifted options for treatment from surgical resection to combination antibiotic therapy [1]. While PCR analysis targeting the insertion sequence IS*2404* has evolved into the gold standard for laboratory diagnosis of BU, this test is only available at a few reference centres. Therefore, the diagnosis of BU is currently often based on clinical findings and antibiotic therapy is started before laboratory diagnostic results can be obtained. BU has a wide range of clinical manifestations including non-ulcerative forms such as subcutaneous nodules or papules, plaques and oedema, which may progress to chronic ulcerative lesions. Due to this diversity of disease presentations the accuracy of clinical diagnosis is limited [1, 3–5] and thus a significant proportion of patients reporting with skin lesions may not receive adequate treatment. This includes cases of cutaneous tuberculosis which may be misdiagnosed as BU and thus receive the recommended eight week course of Streptomycin/Rifampicin combination chemotherapy for BU [5], which is much too short for the treatment of tuberculosis. As for IS*2404* PCR, two of the other three currently applied methods for laboratory reconfirmation of BU—histopathology and cultivation of the extremely slow-growing mycobacteria—equally require expensive equipment and expertise [4, 6–8] not accessible at peripheral health facilities. The only available point-of-care diagnostic test, direct-smear examination by microscopy for the detection of acid fast bacilli (AFB), has limited sensitivity and specificity [6]. Hence, one of the major research priorities for BU is the development of a fast, low-tech, sensitive and specific point-of-care diagnostic test, which can be directly implemented at peripheral health centres.

The development of a specific point-of-care diagnostic test for the detection of *M. ulcerans* is complicated by the broad antigenic cross-reactivity among the various mycobacterial species. Serological approaches targeting the few *M. ulcerans*-specific antigens identified, turned out to be not suitable for differentiation between BU patients and exposed control individuals, as both groups may or may not exhibit serum IgG titers against these antigens [9–11].

In recent years, point-of-care tests in the form of antigen capture assays have successfully been developed for tropical infectious diseases [12]. Extensive studies focussing on rapid diagnostic tests for malaria [13–17] have paved the way for the development of antigen capture based assays for other diseases such as dengue fever [18, 19], hepatitis C [20, 21] or visceral leishmaniasis [22] to name but a few.

In the present work we aimed at the identification of targets for the development of an antigen capture test for the diagnosis of *M. ulcerans* infection by using a proteomics approach.

## Materials and Methods

### Ethics statement

Ethical clearance for the analysis of clinical specimens was obtained from the Cameroon National Ethics Committee (N°172/CNE/SE/201) and the Ethics Committee of Basel (EKBB, reference no. 53/11). Immunization of mice for the generation of monoclonal antibodies was performed in strict accordance with the rules and regulations for the protection of animal rights (“Tierschutzverordnung”) of the Swiss “Bundesamt für Veterinärwesen”.

All animal infection experiments performed were approved by the animal welfare committee of the Canton of Vaud (authorization number 2261) and were conducted in compliance with the Swiss animal protection law under BSL-3 conditions.

### Mycobacterial strains

In this study we analyzed *M. ulcerans* isolates from Ghana (NM20/02), Côte d’Ivoire (ITM 940511), Togo (ITM 970680), China (ITM 98912), Japan (ITM 8756) and Australia (JS5147) as well as additional mycobacterial strains including *M. abscessus* (ATCC 19977), *M. avium* (MAC101), *M. chelonae* (DSM 43804), *M. fortuitum* (ATCC 49403), *M. gordonae* (Pasteur 14021.001), *M. haemophilum* (ATCC 29548), *M. intracellulare* (clinical isolate), *M. kansasii* (NCTC 10268), *M. lentiflavum* (clinical isolate), *M. malmoense* (NCTC 11298), *M. marinum* (ATCC 927), *M. scrofulaceum* (Pasteur 14022.0031), *M. simiae* (clinical isolate) *M. smegmatis* (Pasteur 14133.0001), *M. terrae* (clinical isolate), *M. xenopi*, *M. bovis* (ATCC 35734) and *M. tuberculosis* (Pasteur 14001.0001). *M. ulcerans* strains were grown in BacT/Alert culture bottles supplemented with enrichment medium according to the manufacturer’s protocol (bioMérieux).

### Preparation of protein lysates

For the preparation of *M. ulcerans* protein lysates, bacteria (5 ml of culture, OD_600_~1) were washed in PBS, heat-inactivated at 95°C for 35 min, centrifuged at 10′000 × g for 10 min and resuspended in 400 μl lysis buffer (PBS containing 5% SDS, 1 mM phenylmethylsulfonyl fluoride (PMSF) and a protease inhibitor cocktail (complete mini, Roche)). The mix was transferred to lysing tubes (Precellys) and homogenized using a mechanical bead beater device (Precellys 24, Bertin Technologies) twice at 6′800 rpm for 30 s. Beads and non-lysed cells were removed by centrifugation at 10′000 × g for 10 min. The preparation of lysates of other mycobacterial species was described previously [9].

### 2D gel electrophoresis of *M. ulcerans* lysates

90 μg of trichloroacetic acid (TCA) precipitated *M. ulcerans* (NM20/02) protein lysate was resuspended in rehydration buffer (8 M urea, 2% 3-[(3-Cholamidopropyl)-dimethylammonio]-1-propanesulfonate (CHAPS), 0.5% (v/v) ZOOM Carrier Ampholytes (Invitrogen), 0.002% bromophenol blue and 0.4% dithioerythritol (DTE)). The mix was incubated with a 3–10 pH gradient strip (ZOOM Strip; Invitrogen) over night (ON) at room temperature (RT). First-dimension isoelectric focusing (IEF) was performed on a ZOOM IPG runner (Invitrogen) using a step voltage protocol (175 V for 15 min, 175–2000 V for 45 min, 2000 V for 2 h). After IEF, the strips were incubated for 15 min with equilibration buffer (6 M urea, 50 mM Tris pH 8.8, 30% glycerol, 2% SDS, 30 mM DTE) followed by a 15 min incubation period with alkylating solution (6 M urea, 50 mM Tris (pH 8.8), 30% glycerol, 2% SDS, 0.23 M iodacetamide). Second-dimension gel electrophoresis was performed at 200 V for 35 min using a 10% NuPAGE Novex Bis-Tris ZOOM Gel (Invitrogen). The gel was stained with Coomassie blue (Invitrogen).

### In-gel tryptic digestion of proteins

All Coomassie stained protein spots were selected for mass spectrometry analysis. Spots were excised from the 2D gel, placed in a low-binding microcentrifuge tube and destained in 0.1 M ammonium bicarbonate / 30% acetonitrile at 30°C. Gel spots were dried in a SpeedVac concentrator and digested with 4 μl of 10 μg/ml trypsin (trypsin porcine, Roche Applied Science) ON at 37°C. Peptides were extracted from gel pieces with 4 μl of 0.3% trifluoroacetic adic (TFA) / 50% acetonitrile. The samples were desalted and concentrated using ZipTipC18 tips (Millipore). Eluted peptides were loaded onto a MALDI target.

### Mass spectrometry (MS) and data analysis

MS analysis was performed using a MALDI-TOF mass spectrometer (Bruker ultraflex III TOF/TOF, Bruker Daltonics Inc.) in the reflector mode. 1 μl of tryptic digest and 1 μl of matrix (5 mg/ml α-cyano-4-hydroxycinnamic acid, 50% acetonitrile, 0.1% TFA) were spotted onto a MALDI target (MTP AnchorChip 600/384, Bruker Daltonics) and allowed to co-crystallize at room temperature. Data were processed using FlexAnalysis software (Bruker Daltonics flexAnalysis 2.4). Spectra were smoothed (Sawitzgy Golay algorithm, 0.2 m/z width, 1 cycle), baseline subtracted (median algorithm, 0.8 flatness) and calibrated using trypsin autocleavage or internal standard peptide mass peaks. A monoisotopic peak list was generated from the spectrum using SNAP algorithm and analyzed with BioTools (Bruker Daltonics BioTools 3.0). Peptide mass fingerprinting searches were performed using the Aldente search engine on the Expasy server.

### Recombinant expression of full length MUL_3720 and truncated MUL_3720

The full length MUL_3720 (aa 1–207) and a truncated version of this protein lacking the lectin domain (aa 115–207) were recombinantly expressed in *Escherichia coli* BL21 Star (DE3, Invitrogen) as N-terminal hexahistidin-tagged fusion proteins. Briefly, PCR was performed on a pUC57 vector containing the DNA sequence of MUL_3720 generated by gene synthesis (Genscript), including *Nde*I and *Not*I restriction sites. The amplified sequences were inserted into a TOPO-TA cloning vector using the TOPO Cloning Kit and introduced into *E. coli* (Top 10, Invitrogen). The vector was digested with *Nde*I and *Not*I (New England Biolabs) and the sequence was ligated into a pET28a expression vector using the Rapid DNA Ligation Kit (Roche). *E. coli* BL21 Star (DE3, Invitrogen) were grown in Luria-Bertani (LB) medium until an OD_600_ of ~0.5. Protein expression was induced by addition of isopropyl thiogalactoside (IPTG) to a final concentration of 1 mM and subsequent incubation for 3 h at 37°C. Bacteria were lysed by sonication and His-tagged proteins were purified by nickel-nitrilotriacetic acid (Ni-NTA) chromatography.

### Construction of a MUL_3720 overexpressing *M. ulcerans* strain

*MUL_3720* was amplified from genomic *M. ulcerans* DNA and cloned into TOPO vector using *Nde*I and *Sca*I restriction sites. Electrocompetent *E. coli* TOP10 cells (Invitrogen) were transformed with *TOPO::MUL_3720* vectors and spread onto LB-Ampicillin (50 μg/ml) agar. Plasmid DNA of the mutant colonies was prepared and inserts with correct size and sequence were excised from TOPO by *Nde*I/*Sca*I and ligated into the mycobacterial vector pSD5. Chemically competent *E. coli* TOP10 were transformed with *pSD5::MUL_3720* and grown on LB-Kanamycin (50 μg/ml) plates. Plasmid DNA was prepared and the presence of the insert was confirmed.

*M. ulcerans* strain NM20/02 was grown in BacT bottles (bioMérieux) containing enrichment medium (bioMérieux). Bacteria were harvested and washed twice with 10% glycerol or distilled water. Competent *M. ulcerans* bacteria were electroporated (2.5 kV, 1000 Ohm, 25 μF) with varying amounts (50–1000 ng) of DNA, transferred to MGIT-OADC medium (BD) and grown under non-selective conditions for 36 hours at 30°C. After the recovery phase, bacteria were spread on 7H10-Kanamycin (25 μg/ml) agar and incubated for several months at 30°C. Colonies were picked and regrown on selective agar and in BacT bottles (bioMérieux) in order to prepare lysates and stocks.

### Generation of antibodies against MUL_3720

For the preparation of mAbs, mice were immunized two times intraperitoneally with 40 μg of recombinant full length MUL_3720 (aa 1–207) emulsified in Immune Easy adjuvant (Qiagen). Two weeks after the second immunization serum antibody titres against MUL_3720 (aa 1–207) as well as against the truncated MUL_3720 (aa 115–207) were determined by ELISA. Based on these results one BALB/c mouse was selected to receive a final intraperitoneal injection of 40 μg of recombinant MUL_3720 (aa 1–207) without adjuvant. Three days after this last booster dose, hybridoma cell lines were generated as described previously [23]. Briefly, the spleen of the selected mouse was removed and the spleen cells were fused with mouse myeloma cells (PAI cells). After a few days, cell culture supernatants were tested for the presence of anti-MUL_3720 (aa 1–207) as well as anti-MUL_3720 (aa 115–207) antibodies. Positive cell lines were cloned by limiting dilution and expanded. MAbs were purified using HiTrap rProtein A column (Amersham Biosciences). Two individual fusion experiments resulted in 24 and 17 MUL_3720-ELISA positive B-cell hybridoma cell lines, respectively. Of these, a total of 5 B-cell hybridoma cell lines (JD3.1, JD3.2, JD3.3, JD3.4, JD3.6, JD3.7) were successfully cloned and expanded for antibody production.

Rabbit polyclonal antibodies were generated and affinity purified by Eurogentech. New Zealand white Rabbits were injected intramuscularly with 20 μg recombinant MUL_3720 (aa 1–27) with Sigma Adjuvant System (SZ3398) or Imject Alum (SZ3403) on day 0, 14, 28 and 56. Total IgG was purified from antiserum collected on day 66 by protein A affinity chromatography.

### ELISA

96-well Immulon microtiter plates (Thermo Scientific) were coated with 1 μg recombinant MUL_3720 (aa 1–207) or MUL_3720 (aa 115–207) per well in 100 μl PBS and incubated ON at 4°C. Plates were washed three times with washing buffer (2.5% Tween 20 in dH_2_O) and blocked with 5% non-fat dry milk in PBS containing 0.1% Tween for 1 h at 37°C. After washing as described above, 100 μl of the primary antibody (mAbs or hybridoma supernatant) was added and incubated for 2 h at 37°C. Following an additional washing step, 100 μl of 1:30′000 diluted goat anti-mouse IgG (γ-chain specific) antibodies coupled to alkaline phosphatase (SouthernBiotech) was added to each well and incubated for 1 h at 37°C. Plates were washed and 100 μl/well of phosphatase substrate solution (1 mg/ml p-Nitrophenyl phosphate in substrate buffer) was added and incubated for 1 h at 37°C. Absorbance at 405 nm was measured with a microplate reader (Tecan Sunrise).

### Western blot analysis

2 μg of mycobacterial protein lysates per lane were separated on NuPAGE Novex 4–12% Bis-Tris ZOOM Gels (Invitrogen) using NuPAGE MES SDS Running Buffer (Invitrogen) under reducing conditions. After electrophoresis proteins were transferred onto nitrocellulose membranes using an iBlot gel transfer device (Invitrogen). Membranes were blocked with blocking buffer (5% non-fat dry milk in PBS) ON at 4°C. Membranes were then incubated in blocking buffer containing anti-MUL_3720 IgG (mouse mAbs JD3.2, JD3.4 or rabbit polyclonal IgG SZ3398) or mouse mAb DD3.7 (specific for a conserved mycobacterial protein) serving as loading control for 1 h at RT. After washing, membranes were incubated with secondary goat anti-mouse IgG (γ-chain specific) (HRP, SouthernBiotech) or goat anti-rabbit IgG (Fc fragment specific) (HRP, Milan) for 45 min at RT. After washing, bands were visualized by chemiluminescence using the ECL Western Blotting substrate (Pierce).

### Histology

Immunohistochemical analysis was performed on tissue or punch biopsies from different IS*2404* qPCR reconfirmed patients. Tissue or punch biopsies of BU patients were removed aseptically and immediately fixed in 10% neutral buffered formalin for 24 hours. Afterwards the tissue was embedded into paraffin, cut into 5 μm thin sections and transferred onto microscopy glass slides. Immunohistochemical staining of the sections was performed after deparaffinisation, rehydration and antigen retrieval with citrate-pretreatment according to standard protocols [24]. Inactivation of endogenous peroxidase as well as prevention of unspecific binding was achieved by incubation in PBS containing 0.3% hydrogen peroxide and 1.5% horse serum for 20 min. Primary anti-MUL_3720 IgG was diluted in PBS containing 0.1% Tween-20 and added to the slides for 1 h at RT or ON at 4°C. After incubation with biotin-conjugated horse anti-mouse IgG, slides were stained using the Vector ABC and NovaRED system. Sections were counterstained with haematoxylin.

JD3.4 and JD3.2 showed a comparable staining in intensity, specificity and sensitivity. JD3.2 gave a slightly lower unspecific background staining of the surrounding tissue and was used for IHC analysis.

### Immune fluorescence assay (IFA)

IFA was carried out as described previously [25]. Briefly, a pellet of *M. ulcerans* bacteria (OD_600_~0.6) was resuspended in 1.5% low-melting agarose (BioWhittaker Lonza, Basel Switzerland) and transferred to cryomodules (Applied BioSystems). Agarose blocks were embedded into paraffin, cut in 3 μm sections and transferred onto microscopy glass slides (Thermo Scientific). Bacteria were stained with mAb JD3.2 and Alexa fluor488 (Invitrogen) conjugated goat anti-mouse IgG and mounted in ProLong Gold anti-fade reagent containing 4′,6-Diamidino-2-phenylindole (DAPI; Invitrogen).

### Mycobacterial protein fragment complementation

The system described for investigating protein interactions by the functional reconstitution of a murine dehydrofolate reductase domain in *M. tuberculosis* [26] was modified here for use in *M. ulcerans.* N-terminal and C-terminal fusions of the bait domains to full length MUL_3720 were constructed using pUAB400 or pUAB200. Cloning was facilitated by the *Mfe*I and *Cla*I restriction sites in the pUAB multiple cloning sites. All cloning was performed using *E. coli* DH10B and confirmed by Sanger sequencing. Plasmids were extracted from *E. coli* using mini-prep columns (Qiagen) and plasmid DNA was used to transform *M. smegmatis* MC^2^155 by electroporation as previously described [27].

*M. ulcerans* Agy99 genomic DNA libraries were prepared by partial *Aci*I digestion. Digested DNA between 500 bp and 3 Kbp was purified using a gel purification kit (Qiagen), ligated into *Cla*I digested pUAB300 and used to transform *E. coli* DH10B. A number of colonies were randomly selected for PCR using primers F102 (5′-agaaccaccacgaggagctcat-3′) and R102 (5′-tgatgcctggcagtcgatcgta-3′) that flank the multiple cloning site on the vector to check for insertions containing inserts within the desired size range [26]. Approximately 2 × 10^5^ clones were subsequently collected and cultured in LB ON. Plasmid DNA maxi-preps were performed on ON cultures according to the manufacturer’s instructions (Sigma-Aldrich).

Bacteria co-transformed with plasmids containing interacting, complementary mDHFR fragments were selected on 7H11 kanamycin (25 μg/ml) and hygromycin (50 μg/ml) plates. Colonies were patched onto 7H11 kanamycin-hygromycin-trimethoprim plates and colonies resistant to trimethoprim were selected for PCR. Using primers F102 and R102, PCR products were sequenced. The sequences were used to perform BLAST against the *M. ulcerans* Agy99 genome. Inserts containing open reading frames in the incorrect orientation were discarded. Also removed were inserts that matched non-coding genomic DNA or the dehydrofolate reductase from *M. ulcerans*.

### Bacteria and mouse foot pad infection model

*M. ulcerans* strain S1013 used for experimental infection of mice was isolated in 2010 from the ulcerative lesion of a Cameroonian BU patient [28]. Bacteria were cultivated in Bac/T medium for 6 weeks, recovered by centrifugation and a stock suspension in sterile PBS of 125 mg/ml wet weight was prepared. 30 μl of a 1:1000 dilution of the stock solution was injected subcutaneously into the left hind foot pad of 14 week old female BALB/c mice. On day 87 after infection, mice were euthanized and foot pads were aseptically removed. Foot pads were dipped into 70% ethanol, dried under the laminar flow, cut into four pieces with a scalpel and transferred to reinforced hard tissue grinding tubes (MK28-R, Precellys) containing 750 μl of Bac/T medium (bioMérieux). Tissue homogenization was performed with a Precellys 24-dual tissue homogenizer (3 × 20 s at 5000 rpm with 30 s break). After transferring the supernatant to a fresh tube, the residual tissue remains were homogenized a second time in 750 μl of Bac/T medium. Tissue lysates were pooled and stored at -80°C until further use. 500 μl of thawed tissue lysate was transferred into tough microorganism lysis tubes (VK05–2ml, Precellys), inactivated for 1 h at 85°C and centrifuged at 17′000 × g for 5 min. The pellet was resuspended in 250 μl PBS containing protease inhibitors (Roche, EDTA—free) and cells were disrupted with Precellys 24-dual tissue homogenizer (2 × 30s at 6800rpm with 1 min break in between). Lysates were cleared by centrifugation and tested by ELISA.

### Antigen capture assay

Nunc-Immuno Maxisorp 96-well plates (Thermo Scientific) were coated with 10 μg/ml JD3.4 mAb (50 μl per well) in PBS and incubated ON at 4°C. Plates were washed three times with washing buffer (2.5% Tween 20 in dH_2_O) prior to incubation with blocking buffer (5% non-fat dry milk in PBS) for 2 h at RT. After washing as described above, 50 μl of different dilutions of the purified recombinant full length MUL_3720, *M. ulcerans* lysate (NM20/02), or lysates from *M. ulcerans* infected tissue samples in PBS were added and incubated for 2 h at RT. Following an additional washing step, 50 μl anti-MUL_3720 rabbit IgG (5 μg/ml) in blocking buffer with detergent (0.5% non-fat dry milk in PBS containing 0.05% Tween 20) was added and incubated for 2 h at RT. After washing as described above, 50 μl goat anti-rabbit IgG coupled to horseradish-peroxidase (Milan) diluted 1:10′000 in blocking buffer with detergent was added and incubated for 1 h at RT. Plates were washed and TMB peroxidase substrate solution was added. After 10 min the reaction was stopped with 2 M sulfuric acid and absorbance was measured at 450 nm with a microplate reader (Tecan Sunrise).

## Results

### Identification of MUL_3720

For identification of suitable proteins that could be used as targets in diagnostic test formats, an *M. ulcerans* whole protein lysate was analysed by 2D gel electrophoresis (S1 Fig.). In total, 384 protein spots were detected, processed and subsequently subjected to MALDI-TOF-MS. Among the 384 spots, 118 peptide fragments were identified and attributed to 36 different genes. In order to select for proteins without orthologs in *M. tuberculosis*, *M. bovis* or *M. leprae*, a BLAST search against the Uniprot database was performed for all 36 proteins, resulting in the identification of three potential targets (MUL_3720, MUL_0343 and MUL_4023) suitable for a selective antigen capture assay. However, MUL_0343 and MUL_4023 presented very weak protein spots in the 2D gel, while MUL_3720 showed a high expression level and was therefore selected for further analysis.

**Fig 1:**
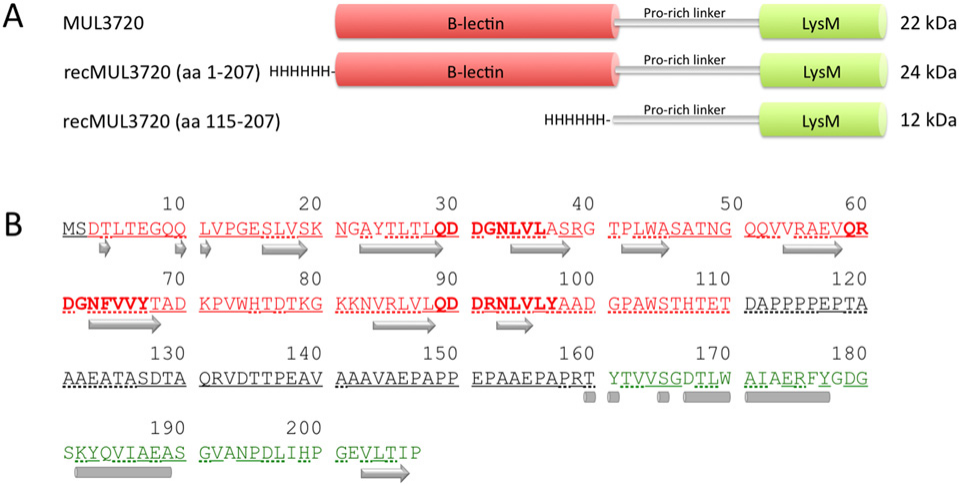
Schematic presentation of MUL_3720. (A) The MUL_3720 protein consists of a N-terminal B-lectin domain and a C-terminal LysM domain linked by a proline rich sequence. MUL_3720 was recombinantly expressed in *E. coli* either as full length (aa 1–207) or truncated protein (aa 115–207) with a N-terminal hexa-histidine-tag. (B) The predicted amino acid sequence is shown with residues of the B-lectin and the LysM domain marked in red and green, respectively. The consensus sequence motifs QXDXNXVXY involved in alpha-D-mannose recognition are indicated in bold. Strands (arrows), helixes (bars), exposed (underlined) and buried (dotted underlined) regions were predicted with PredictProtein.

The 624 bp *MUL_3720* gene encodes a protein of 207 amino acids, with a molecular mass of 22 kDa. MUL_3720 is predicted to possess an N-terminal bulb-type mannose-specific lectin domain and a C-terminal peptidoglycan-binding Lysin Motif (LysM) linked by a proline-rich sequence (Fig. 1). Database comparisons revealed the presence of orthologs with a similar domain organisation in *M. abscessus* (MAB_2373), *M. avium*, *M. colombiense*, *M. fortuitum*, *M. kansasii* (MKAN_05370), *M. marinum* (MMAR_3773), *M. smegmatis* (MSMEG_3662) and *M. xenopi*. The *M. marinum* ortholog displayed a sequence identity of 99% (S2 Fig.).

### Generation and characterization of anti-MUL_3720 antibodies

**Fig 2:**
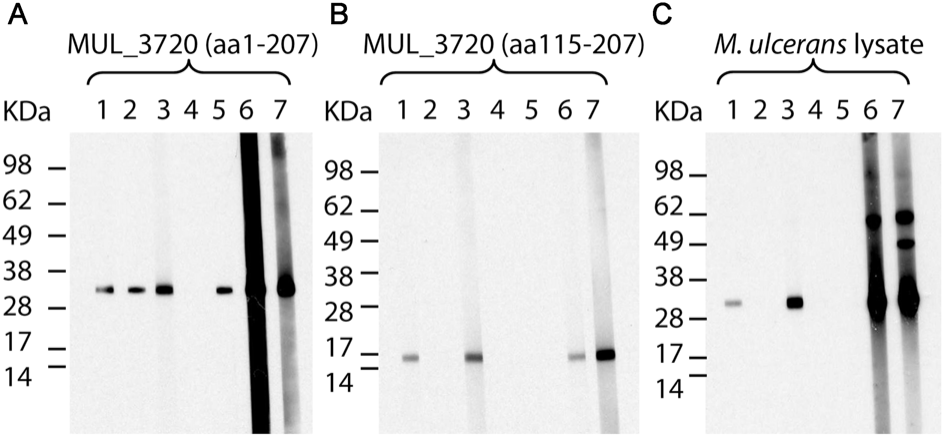
Reactivity of MUL_3720-specific antibodies. Mouse mAbs JD3.2 (1), JD3.3 (2), JD3.4 (3), JD3.6 (4) and JD3.7 (5) and rabbit polyclonal IgG SZ3398 (6) and SZ3403 (7) were tested for their reactivity with MUL_3720 (aa 1–207) (**A**), truncated MUL_3720 (aa 115–207) (**B**) and the endogenous MUL_3720 in a total protein lysate of *M. ulcerans* strain NM20/02 (**C**) by Western Blot analysis.

For the generation of antibodies against MUL_3720, required for the detection of the protein in diagnostic assays, we immunized mice and rabbits with the full length protein, recombinantly expressed as a His-tagged fusion protein (predicted molecular mass 24 kDa) in *E. coli* BL21. Hybridoma cell lines producing antibodies against different epitopes of the protein were identified by analyzing their reactivity against MUL_3720 (aa 1–207) as well as the truncated version of MUL_3720 (aa 115–207), lacking the lectin domain and consisting only of the LysM motif and the proline-rich sequence. Five mAbs (JD3.2, JD3.3, JD3.4, JD3.6 and JD3.7), all of them mouse IgG1(κ) isotype, were generated, purified and further characterized. All the mAbs recognized recombinant full length MUL_3720 (aa 1–207) in ELISA. While all antibodies except for JD3.6 recognized recombinant full length MUL_3720 (aa 1–207) in Western Blot analysis (Fig. 2A), only JD3.2 and JD3.4 also reacted with recombinant truncated MUL_3720 (aa 115–207) (Fig. 2B) and the endogenous protein in *M. ulcerans* lysates (Fig. 2C) (S1 Table).

### Reactivity of the generated antibodies with lysates of different *M. ulcerans* strains and other mycobacteria

**Fig 3:**
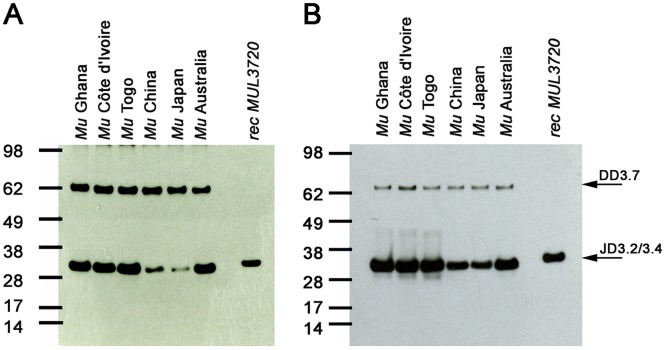
MUL_3720 is expressed by *M. ulcerans* strains of various geographic origin. Total protein lysates of *M. ulcerans* strains from Ghana, Côte d’Ivoire, Togo, China, Japan and Australia were analyzed by Western Blot. Detection with mAbs JD3.2 (**A**) and JD3.4 (**B**) demonstrates expression of MUL_3720 in strains from different geographical regions, while staining with mAb DD3.7 served as a loading control. MUL_3720 expression levels are lower in *M. ulcerans* strains belonging to the ancestral (China, Japan) as compared to those of the classical lineage (Africa and Australia).

The ability of mouse mAbs JD3.2 and JD3.4 to detect endogenous MUL_3720 in lysates of *M. ulcerans* strains from different geographical regions was examined by Western Blot analysis. While the protein was recognized by JD3.2 and JD3.4 in all *M. ulcerans* strains, isolates belonging to the classical *M. ulcerans* lineage (Ghana, Côte d’Ivoire, Togo and Australia) showed higher expression levels as compared to isolates belonging to the ancestral lineage (China and Japan) (Fig. 3).

**Fig 4:**
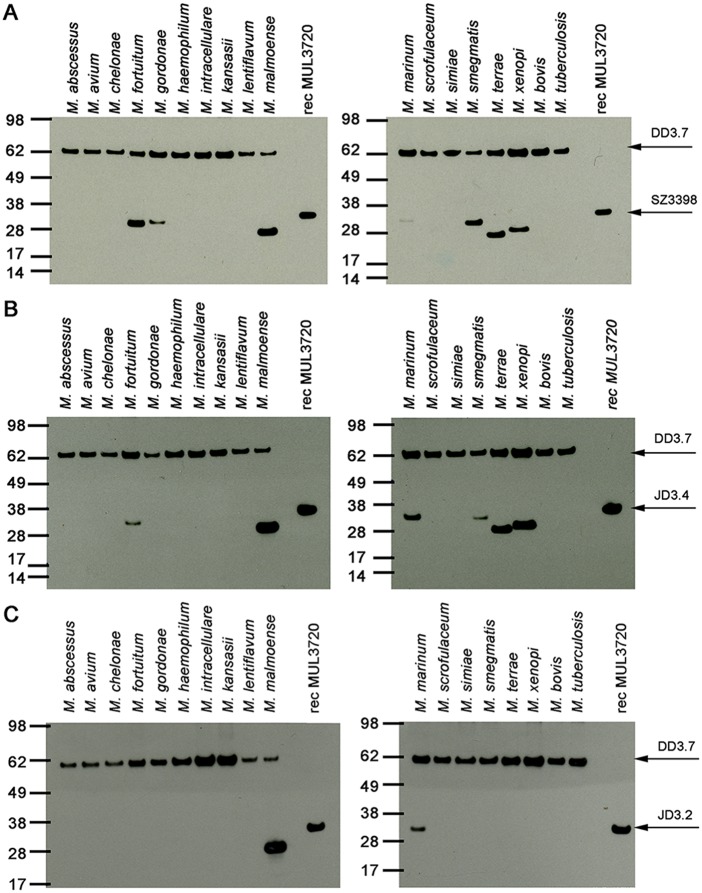
Analysis of mycobacterial lysates for the presence of MUL_3720 orthologs. Total protein lysates of different mycobacterial strains were analyzed by Western Blot. MAb DD3.7 served as a loading control. (A) Detection with pAb SZ3398 demonstrates expression of MUL_3720 orthologs in some of the mycobacteria tested, but not in *M. tuberculosis* and *M. bovis*. (B) MUL_3720 orthologs in additional mycobacterial species were detected with JD3.4, indicating the recognition of different protein epitopes. (C) Analysis with mAb JD3.2 led to the detection of MUL_3720 orthologs in *M. malmoense* and *M. marinum*.

Interspecies cross-reactivity of MUL_3720 was determined by Western Blot analysis with lysates of a range of different mycobacterial species (Fig. 4). In accordance with the BLAST search for MUL_3720 orthologs (S2 Fig.), rabbit polyclonal anti-MUL_3720 IgG reacted with proteins in lysates of *M. fortuitum*, *M. marinum*, *M. smegmatis* and *M. xenopi*. In agreement with a shorter linker between the lectin and the LysM domains (S2 Fig.), the *M. xenopi* ortholog was detected at a lower molecular weight. The predicted orthologous proteins in *M. abscessus*, *M. avium* and *M. kansasii* were not recognized by the rabbit polyclonal IgG. Furthermore, a protein band was observed in lysates of *M. gordonae*, *M. malmoense* and *M. terrae* for which no sequence information is available (Fig. 4A). The detected proteins in *M. malmoense* and *M. terrae* were slightly smaller than MUL_3720.

Analysis with JD3.4 led to a similar staining pattern among the mycobacterial lysates, except for the protein expressed by *M. gordonae*, which was not recognized (Fig. 4B). JD3.2 only reacted with protein in lysates of *M. malmoense* and *M. marinum*, suggesting that the two mAbs JD3.2 and JD3.4 recognize different epitopes of MUL_3720 (Fig. 4C).

### Localization of MUL_3720

**Fig 5:**
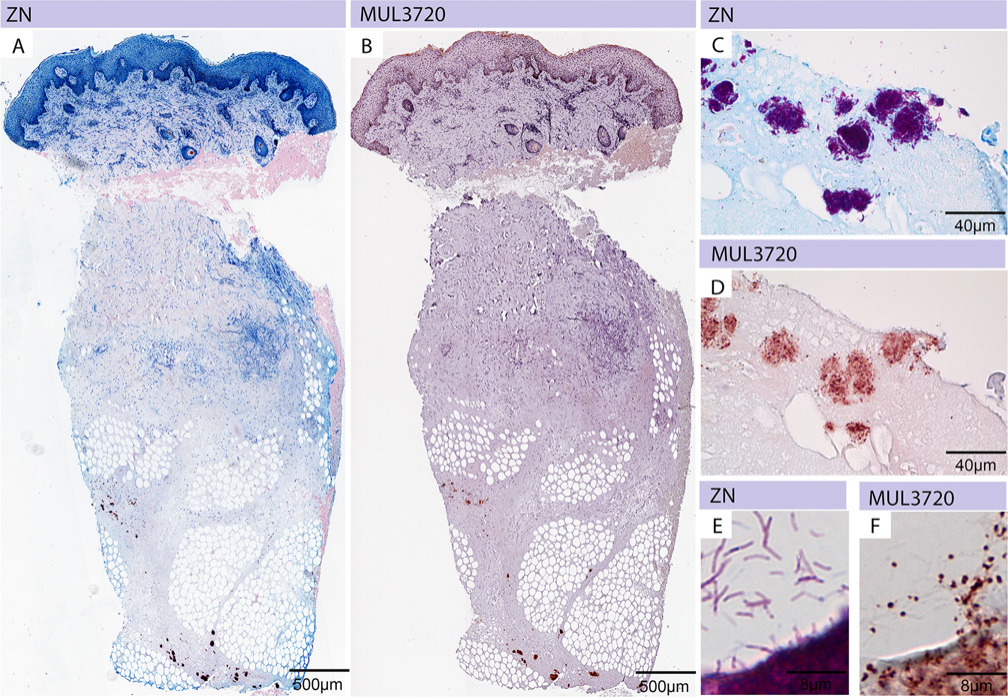
Immunohistochemical analysis of *M. ulcerans* using anti-MUL_3720 antibodies. Histological sections were either stained with carbolfuchsin (counterstain methylenblue) (A, C, E) or with specific monoclonal antibodies (JD3.2) against MUL_3720 (B, D, F). (A) A punch biopsy of a BU patient showing the typical histopathological hallmarks of an active lesion and harbouring large clusters of AFBs inside the subcutaneous tissue (purple clumps) is depicted. (B) Staining of the same tissue specimen with anti-MUL_3720 antibodies. Serial sections were stained by ZN **(C)** and anti-MUL_3720 antibodies **(D)**. While the whole bacterium is stained by ZN **(E)** single dots are detected by using anti-MUL_3720 antibodies **(F)**.

**Fig 6:**
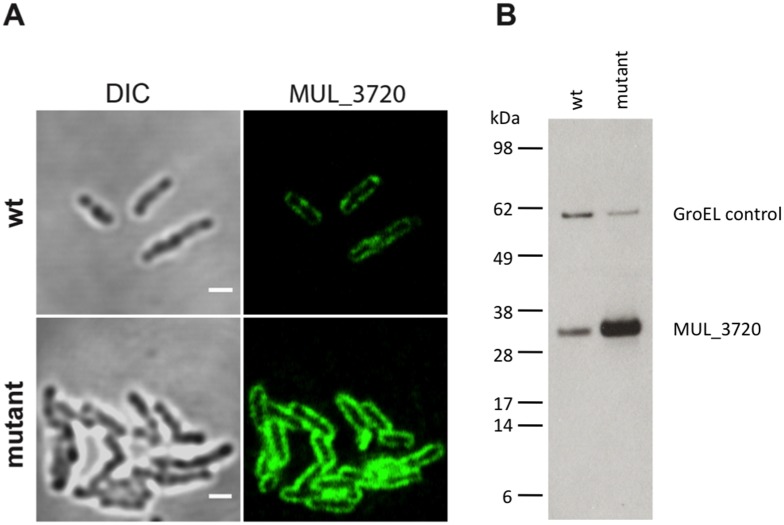
Localization of MUL_3720. Localization of MUL_3720 in the wildtype or overexpressing M. ulcerans strain. (**A**) IF analysis (magnification 1000x) shows that MUL_3720 is homogeneously distributed at the cell surface. Primary JD3.2 anti-MUL_3720 antibody was used at a concentration of 0.1 μg/ml. Bars: 2 μm (**B**) Western Blot analysis of *M. ulcerans* lysates underlines the high expression of MUL_3720 by the mutant strain. As for the IFA analysis, JD3.2 anti-MUL_3720 antibody was used at a concentration of 0.1 μg/ml. A GroEL-antibody was included as loading control.

In order to confirm the expression of MUL_3720 by *M. ulcerans* and the ability of the anti-MUL_3720 mAb JD3.2 to detect the protein *in vivo*, we performed immunohistochemistry and immunofluorescence stainings. *M. ulcerans* could be detected in punch biopsies of human BU patients with the mAbs JD3.2 (Fig. 5) and JD3.4. ZN staining (Fig. 5 A, C, E) and JD3.2 staining (Fig. 5 B, D, F) of serial sections showed identical localization at the same tissue region. AFBs detected by ZN staining in tissue sections revealed a homogeneous staining pattern, whereas immuno-staining with mAb JD3.2 exhibited a heterogeneous staining pattern of the bacteria with intensively stained poles (Fig. 5), indicating a higher expression of the protein in these areas in the natural environment of the bacteria. In contrast, IFA of *in vitro* cultivated bacteria showed a more homogeneous distribution of the protein on the bacterial cell surface. This localization was confirmed in a MUL_3720 overexpressing *M. ulcerans* strain (Fig. 6).

### Investigation of MUL_3720 function

As a first step to begin to understand the role of MUL_3720 we used a bait and prey approach to identify other *M. ulcerans* proteins that interacted with this protein. We employed the mycobacterium-specific protein fragment complementation (M-PFC) system. An M-PFC bait clone using a N-terminal fusion of MUL_3720 and co-transformation with a random library of *M. ulcerans* genomic DNA fragments in pUAB300 (prey) resulted in approximately 150 trimethoprim resistant colonies. Subsequent clones were patched and screened using primers F102 and R102 to determine the identity of the DNA sequence present in pUAB300 (Table 1). Multiple independent clones were identified for sequences encoding DesA1 (MUL_0445) and a PE-PGRS protein (MUL_0572), together with 13 other single-hit CDS, including an interaction with MUL_3720 itself. Many of the putative interacting proteins had a predicted cell wall location or role in cell wall biosynthesis, in line with the localization data for MUL_3720 revealed by mAb staining (Table 1 and Fig. 6). No interacting proteins were identified using the C-terminal MUL_3720 bait fusion, consistent with the predicted cell wall location for this domain of MUL_3720. The M-PFC detects cytoplasmic protein-protein interactions only [26].

**Table 1:** List of proteins interacting with the N-terminus of MUL_3720 as determined by M-PFC assay.

**Locus Tag**	**Product description**	**Predicted Length (aa)**	**Interacting regions (aa positions) ^1^**	**Predicted location**	**No. clones ^2^ identified**
MUL_0445	Desaturase, DesA1	339	1–84	CYT	3
MUL_0572	PE-PGRS family protein	890	360–478	INT	2
MUL_2853	PE-PGRS family protein	403	1–236	INT	1
MUL_3720	Conserved protein	208	179–207	CYT	1
MUL_4703	Conserved hypothetical protein	571	1–251	CYT	1
MUL_4714	Conserved integral membrane protein	464	380–463	INT	1
MUL_1239	Transmembrane ATP-binding protein ABC transporter	691	570–690	INT	1
MUL_2018	Phenolpthiocerol synthesis type-I polyketide synthase, PpsB	1515	1411–1514	INT^3^	1
MUL_2998	Hydrogen peroxide-inducible genes activator, OxyR	315	1–180	CYT	1
MUL_0568	Alpha-D-glucose-1-phosphate thymidylyl-transferase, RmlA	289	9–288	CYT	1
MUL_1214	Conserved transmembrane protein	317	139–316	INT^3^	1
MUL_4825	Conserved protein	69	1–68	CYT^3^	1
MUL_4327	Cutinase, Cut5	232	159–231	SEC	1
MUL_0656	Mannosyltransferase, PimB	384	264–383	CYT	1
MUL_4092	3-ketoacyl-(acyl-carrier-protein) reductase	247	158–246	CYT	1

Notes: ^1^Location is based on BLASTp searches of pUAB300 clones identified as interacting partners and relative to the *M. ulcerans* Agy99 sequence ^2^Number identified indicates the number of independent clones generated with this assay. The predicted location is based on at least one cellular location prediction search using psortB, lipoP, signalP and secretomep. ^3^Detected in the membrane fraction by proteomics (Tafelmeyer ref). CYT: cytoplasm, INT: integral membrane protein, SEC: secreted.

### Development of an antigen capture assay using the generated immunological reagents

**Fig 7:**
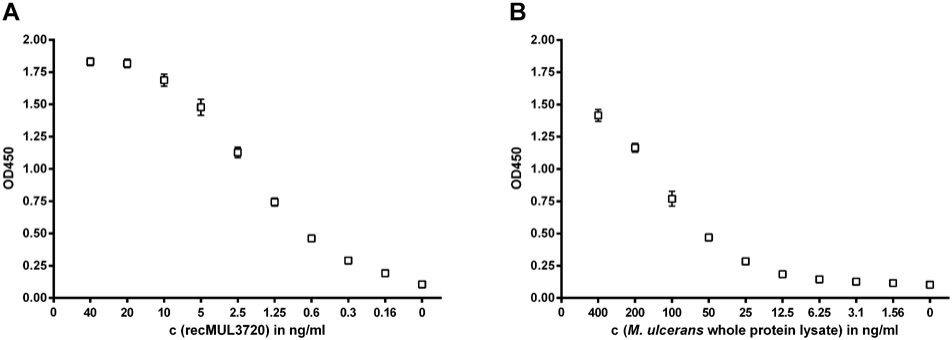
Sandwich ELISA capturing MUL_3720. Antigen capture sandwich ELISAs using mAb JD3.4 in combination with polyclonal anti-MUL_3720 rabbit IgG as capturing and detecting antibodies, respectively, were carried out to detect serial dilutions of recombinant MUL_3720 **(A)** and the endogenous MUL_3720 present in *M. ulcerans* lysates **(B)**.

We analyzed different combinations of the generated mAbs and polyclonal IgG as MUL_3720 capturing and detecting antibodies in an antigen capture sandwich ELISA. The application of mAb JD3.4 as capturing and polyclonal rabbit IgG as detecting reagent enabled a highly sensitive detection of recombinant MUL_3720 (Fig. 7A) and the endogenous protein present in lysates of *in vitro* cultivated *M. ulcerans* (Fig. 7B).

**Fig 8:**
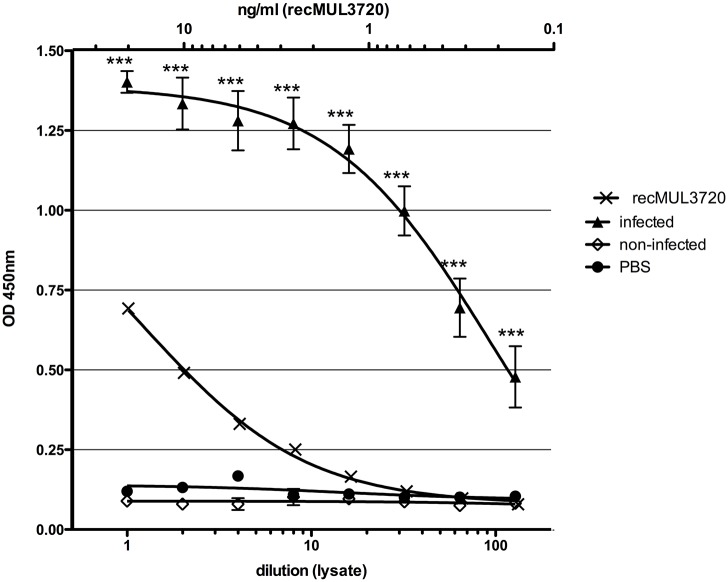
Detection of MUL_3720 in *M. ulcerans* infected mouse foot pads. Antigen capture sandwich ELISAs using mAb JD3.4 in combination with polyclonal anti-MUL_3720 rabbit IgG as capturing and detecting antibodies, respectively, were carried out to detect MUL_3720 in lysates of mouse foot pads infected with *M. ulcerans*. Lysates of non-infected mouse foot pads served as negative control. Purified recombinant MUL_3720 was included as positive control. Asterisks indicate significant differences (P < 0.0001) between mean ODs of infected and non-infected mouse foot pad lysates using a two-tailed Student’s t test.

In order to test if the antigen capture ELISA is able to detect MUL_3720 expressed by bacteria in infected tissue samples, we analyzed lysates of *M. ulcerans* infected mouse foot pads. MUL_3720 could be detected in lysates of all five infected tissue samples analyzed, while only background readouts were obtained for lysates of uninfected foot pads (Fig. 8).

## Discussion

Attempts to develop a diagnostic tool based on serological approaches have been equivocal [9–11], so we decided to focus on direct detection of *M. ulcerans* antigens in BU patient specimens. In the present study, we identified the MUL_3720 protein as a promising target in antigen capture-based diagnostic tests for *M. ulcerans*. Based on 2D gel electrophoretic analyses, MUL_3720 is one of the most highly expressed proteins *in vitro*. The high expression of MUL_3720 is considered an advantage with respect to developing a sensitive antigen detection test for the diagnosis of BU.

While the biological role of MUL_3720 is not known, clues to its function are suggested by its two-domain structure—a conserved bulb-type mannose-binding lectin domain and a Lysin Motif (LysM) domain—predicted to be involved in alpha-D-mannose recognition and in binding to peptidoglycan, respectively. Some bacterial species retain certain proteins attached to peptidoglycan by their LysM domains [29]. We used a mycobacteria-specific two-hybrid system to search for *M. ulcerans* proteins interacting with MUL_3720 and we had hits to a range of proteins known or predicted to be cell-wall associated or involved in cell wall synthesis (Table 1). Many of the interacting proteins—such as DesA1 (Table 1)—are involved in biosynthesis or modification of cell wall molecules. In other mycobacteria, the resulting double bonds from the DesA1-mediated catalysis of a desaturation reaction of saturated alkyl chains that arise during mycolic acid synthesis are required for subsequent position specific modifications such as epoxidation and cyclopropanation of this key cell wall metabolite [30, 31]. *MUL_3720* appears to be arranged in an operon structure with two adjacent putative cell wall-associated protein coding genes (*MUL_3721*, *MUL_3722*). Immunofluorescence stainings of *M. ulcerans* bacilli confirmed the cell wall localization of MUL_3720.

With its cell wall location, the two-domain structure including a mannose-binding N-terminal cytoplasmic component and C-terminal peptidoglycan-binding component, its operon structure and a substantial list of potential interacting proteins, MUL_3720 may be an adaptor protein for multiple cell wall biosynthetic pathways. MUL_3720 might play a role in cell attachment and cell-cell interactions given its presence at the cell surface as revealed by immunofluorescence microscopy and immunohistochemical analyses.

The cell-surface localization of MUL_3720 is an additional advantage with respect to developing a sensitive diagnostic test, since the protein is expected to be easily accessible and detectable in tissue specimens of BU lesions. Potential shedding of the protein from the cell surface may facilitate ready detection in body fluids, which will be examined in future experiments.

Monoclonal and polyclonal antibodies against MUL_3720 were generated for the development of antigen capture assays. These antibodies recognized *in vitro* grown *M. ulcerans* bacilli as well as bacteria in biopsies of human BU patients, proving the expression of MUL_3720 in BU lesions.

Since these antibodies did not react with orthologs of MUL_3720 in other pathogenic mycobacterial species prevalent in the BU endemic regions, prospects for the development of a test with the desired specificity, excluding in particular cutaneous tuberculosis [5], are good. The monoclonal antibodies used for the antigen capture test bind to an epitope on the proline/rich linker and/or the LysM domain. Since the LysM domain is a widespread protein module present in more than 4000 proteins of both prokaryotes and eukaryotes [29], the potential cross-reactivity of the anti-MUL_3720 antibodies with those proteins remains to be analyzed.

Importantly, this capture assay specifically detected MUL_3720 protein in tissue lysates of *M. ulcerans* infected mouse footpads. Furthermore, initial results revealed that MUL_3720 could be detected in swab samples from human BU lesions with a high bacterial burden (manuscript in preparation). Ongoing optimization of the applied reagents as well as the assay format is aiming at the development of a simple test format appropriate for low-resource laboratory settings with suitable test sensitivity.

Antibiotic treatment of BU in its early stages leads in most of the cases to complete healing of the lesions with little or no trauma, whereas treatment at later stages often requires adjunct surgical treatment and is associated with prolonged hospitalization and long-term sequelae. The development of a simple and rapid diagnostic test, whose key elements are provided in the work presented here, will be of immediate benefit to BU patients in rural endemic communities. Clinical findings could directly be reconfirmed by this point-of-care test helping to avoid a false diagnosis and to facilitate a prompt onset of adequate treatment.

## Supporting Information

S1 Fig Two-dimensional gel electrophoretic analysis of *M. ulcerans* proteins. 2D gel of *M. ulcerans* total protein lysate run on a pH3-10 first dimension IPG strip and subsequently run on a 10% SDS-PAGE gel. Coomassie stained protein spots were excised, in-gel digested with trypsin, and subjected to MALDI-TOF MS. Identified proteins are marked as green dots and labelled with the corresponding accession number. Red dots represent proteins that could not be identified. The spots representing MUL_3720 are indicated with an arrow.
(TIF)

S2 Fig Sequence alignment of MUL_3720 and orthologs in other mycobacterial species. Multiple sequence alignment of *M. ulcerans* MUL_3720 and its orthologs in *M. kansasii*, *M. abscessus*, *M. colombiense*, *M. fortuitum*, *M. smegmatis*, *M. marinum*, *M. avium* and *M. xenopi*.
(TIF)

S1 Table Specificity of anti-MUL_3720 mouse mAbs and rabbit polyclonal IgG. All antibodies recognized recombinant full length MUL_3720 (aa 1–207), while JD3.2 and JD3.4 as well as the polyclonal IgG also reacted with recombinant truncated MUL_3720 (aa 115–207) and the endogenous protein in *M. ulcerans* lysates.
(TIF)
