# Supplementary figures and images for: Identification of the Mycobacterium ulcerans Protein MUL_3720 as a Promising Target for the Development of a Diagnostic Test for Buruli Ulcer
Source: PLoS Negl Trop Dis. 2015 Feb 10;9(2):e0003477. doi: 10.1371/journal.pntd.0003477 (PMC4344477; doi:10.1371/journal.pntd.0003477)

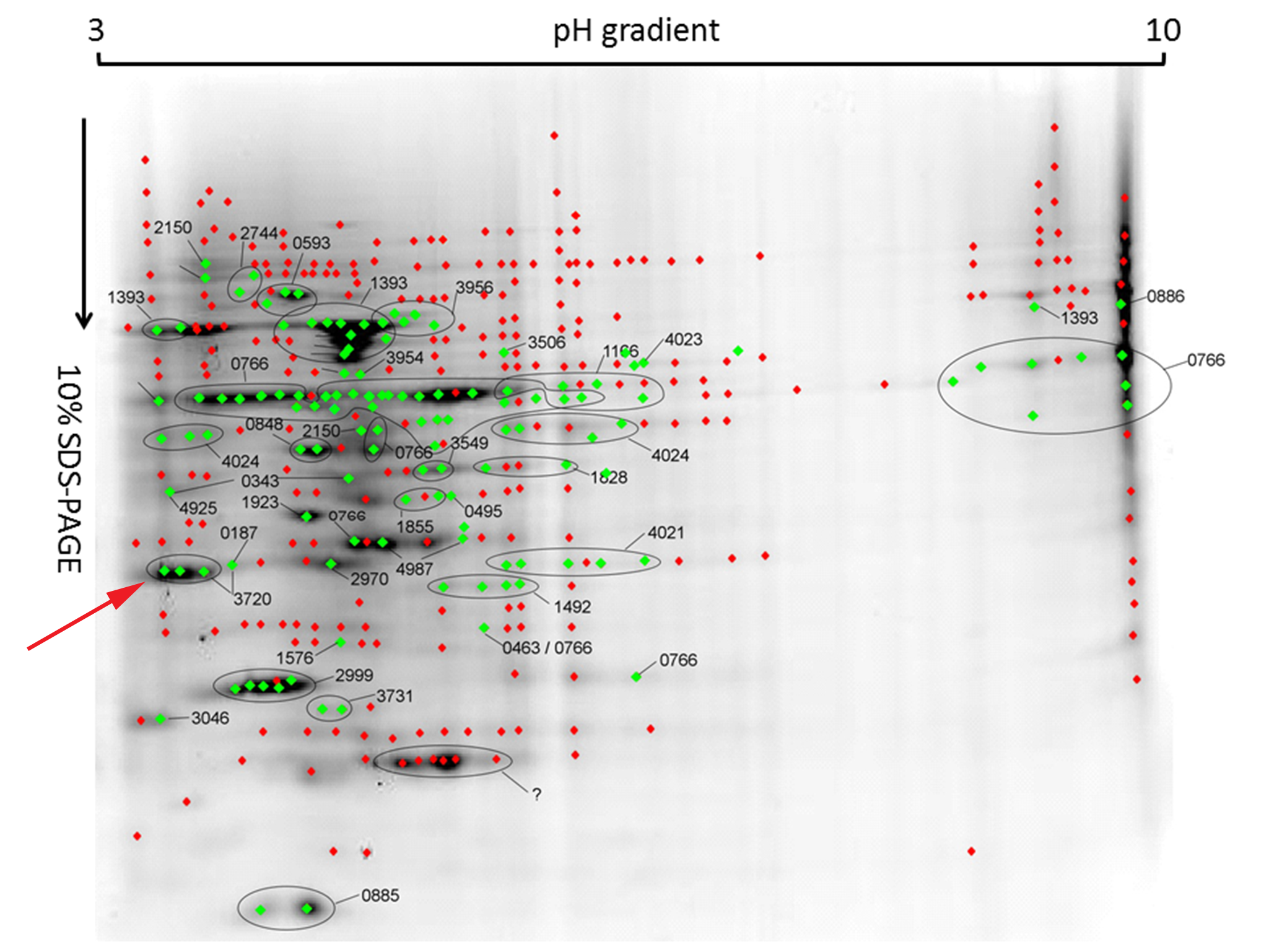

Supplement: S1 Fig — 2D gel of M. ulcerans total protein lysate run on a pH3-10 first dimension IPG strip and subsequently run on a 10% SDS-PAGE gel. Coomassie stained protein spots were excised, in-gel digested with trypsin, and subjected to MALDI-TOF MS. Identified proteins are marked as green dots and labelled with the corresponding accession number. Red dots represent proteins that could not be identified. The spots representing MUL_3720 are indicated with an arrow. (TIF) [file pntd.0003477.s001.tif]

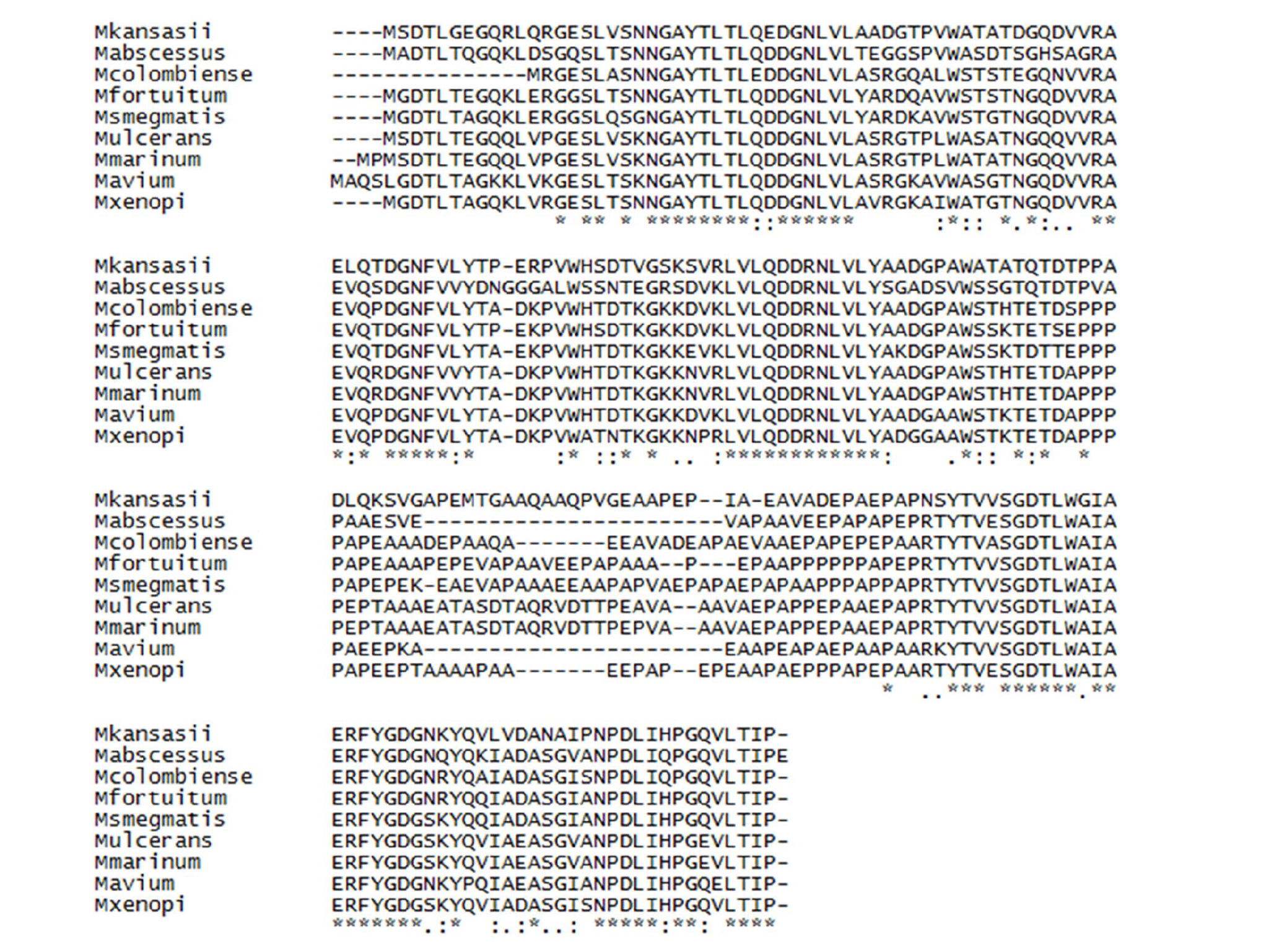

Supplement: S2 Fig — Multiple sequence alignment of M. ulcerans MUL_3720 and its orthologs in M. kansasii, M. abscessus, M. colombiense, M. fortuitum, M. smegmatis, M. marinum, M. avium and M. xenopi. (TIF) [file pntd.0003477.s002.tif]

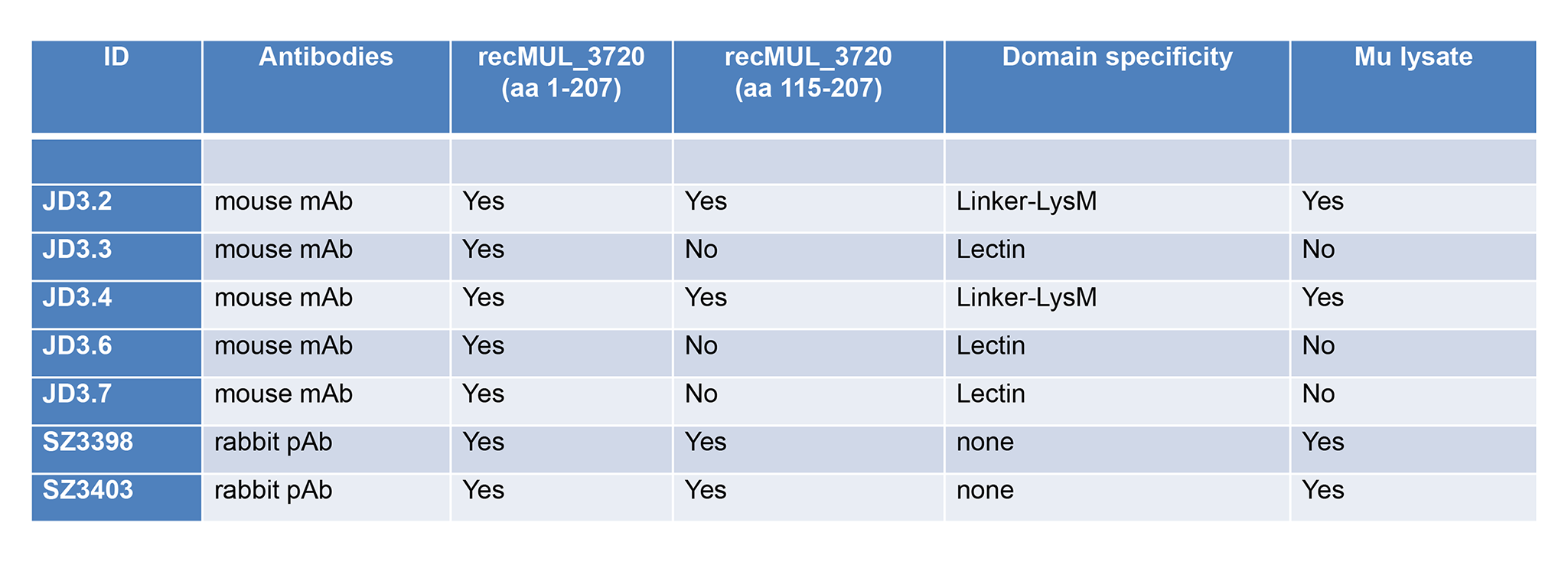

Supplement: S1 Table — All antibodies recognized recombinant full length MUL_3720 (aa 1–207), while JD3.2 and JD3.4 as well as the polyclonal IgG also reacted with recombinant truncated MUL_3720 (aa 115–207) and the endogenous protein in M. ulcerans lysates. (TIF) [file pntd.0003477.s003.tif]
